# Vaginal Estrogen Utilization Among Medicare Beneficiaries With Genitourinary Syndrome of Menopause

**DOI:** 10.1001/jamanetworkopen.2025.49822

**Published:** 2025-12-16

**Authors:** Kelsey Gallo, Chiyuan Amy Zhang, Claire Burton, Neil Kamdar, Ekene A. Enemchukwu

**Affiliations:** 1 Endeavor Health, Glenview, Illinois; 2 Department of Urology, Stanford University School of Medicine, Palo Alto, California; 3 Department of Urology, City of Hope, Duarte, California; 4 Center for Population Health Sciences, Stanford University, Stanford, California; 5 Institute for Healthcare Policy and Innovation, University of Michigan, Ann Arbor; 6 Cecil G. Sheps Center for Health Services Research, University of North Carolina at Chapel Hill, Chapel Hill

## Abstract

**Importance:**

Low-dose vaginal estrogen (VE) is a safe and effective treatment for genitourinary syndrome of menopause (GSM). The frequency of VE prescribing for GSM is unknown.

**Objective:**

To evaluate VE prescriptions among women with GSM and to identify clinical phenotypes associated with VE claims.

**Design, Setting, and Participants:**

This is a retrospective, population-based cohort study of a 20% random sample of Medicare fee-for-service beneficiaries enrolled in Parts A, B, and D from 2006 to 2018. Women aged 66 years and older with a diagnosis indicative of GSM were included. Exclusion criteria included diagnoses of breast and/or endometrial cancer within 6 months of GSM diagnosis. Data analysis was performed from October 2023 to June 2024.

**Exposure:**

Diagnosis indicative of GSM.

**Main Outcomes and Measures:**

The primary outcome was the proportion of women with a VE prescription claim (cream, ring, or tablet) during the follow-up period, from first GSM diagnosis to the end of Medicare enrollment or the study period. To quantify associations between patient characteristics and the likelihood of VE claims, univariable and multivariable logistic regression analyses were conducted.

**Results:**

A total of 1 838 732 women with at least 1 GSM-related diagnosis were identified (median [IQR] age, 74 [69-81] years; median [IQR] follow-up, 8 [4-10] years). VE prescriptions were filled by 165 530 women (9.0%) at a median (IQR) of 15 (2-46) months after diagnosis. Older women (adjusted odds ratio [aOR] for >86 years vs 66-70 years, 0.59; 95% CI, 0.58-0.60) and those with higher Charlson Comorbidity Index scores (aOR for score ≥5 vs 0, 0.67; 95% CI, 0.66-0.69) were less likely to have a VE claim. When comparing GSM symptom groups, women with recurrent urinary tract infections were least likely to have a VE claim (aOR vs local sexual symptoms, 0.54; 95% CI, 0.46-0.64). Women with vulvovaginal symptoms (aOR, 2.70; 95% CI, 2.45-2.97) and GSM multimorbidity (aOR, 15.91; 95% CI, 14.41-17.57) were most likely to have a VE claim.

**Conclusions and Relevance:**

In this large cohort study, 9.0% of female Medicare beneficiaries with a diagnosis indicative of GSM filled a VE prescription. Younger and healthier beneficiaries and those with GSM multimorbidity were more likely to fill a VE prescription. The majority of patients with GSM symptoms, including dyspareunia, vulvovaginal atrophy, and recurrent urinary tract infections, did not fill a VE prescription. Improving patient and practitioner education, revising the diagnostic code schema to capture the full breadth of symptoms, and validating GSM clinical phenotypes will help facilitate care and enhance our understanding of symptoms in research studies.

## Key Points

**Question:**

How prevalent is low-dose vaginal estrogen use for the treatment of genitourinary syndrome of menopause?

**Findings:**

In this cohort study of more than 1 million Medicare beneficiaries, 9.0% of women with a diagnosis indicative of genitourinary syndrome of menopause filled a prescription for vaginal estrogen at a median of 15 months after diagnosis.

**Meaning:**

This study found that the vast majority of patients with symptoms of genitourinary syndrome of menopause did not fill a vaginal estrogen prescription, suggesting that additional research is needed to improve our understanding of symptoms and facilitate care.

## Teaser

This cohort study evaluates vaginal estrogen prescriptions among Medicare beneficiaries with genitourinary syndrome of menopause and evaluates the clinical phenotypes associated with vaginal estrogen claims.

## Introduction

Genitourinary syndrome of menopause (GSM) is a chronic, progressive condition of the vulva, vagina, and lower urinary tract caused by hypoestrogenism.^1^ The term *GSM* was introduced in 2014 to replace the term *vulvovaginal atrophy*.^1^ Specific diagnostic criteria have yet to be established, and the prevalence of the condition is consequently difficult to estimate. However, in a systematic review of available studies, GSM symptoms were present in 45% to 77% of surveyed postmenopausal women.^2,3,4,5,6^ GSM symptoms often begin in perimenopause and become increasingly prevalent with age and time since last menstrual period.^2,7^ Although GSM is most commonly associated with natural menopause, it can also affect women with premature ovarian insufficiency and those who experience surgically or medically induced menopause.^7^

Low-dose vaginal estrogen (VE) therapy is a safe and effective treatment for GSM, with subjective improvement in symptom severity of 60% to 80%.^3,7,8^ The North American Menopause Society recommends VE for women with moderate to severe GSM and for women who do not respond to nonhormonal vaginal lubricants and moisturizers.^7^ Previous estimates of VE use for GSM treatment among US-residing postmenopausal women range from only 2.2% to 4.2%.^9,10^ VE offers a more comprehensive therapeutic approach to GSM management compared with nonprescription topical treatments in a subset of women. In addition to improving vulvovaginal symptoms, VE reduces the risk of future urinary tract infections (UTIs), addressing a broader range of GSM-related conditions.^11,12,13,14,15^ Barriers to VE use are numerous and include cost and misconceptions about clinical indications and safety among patients and practitioners.^2^ Safety concerns are reinforced by product labeling on all estrogen-containing products, despite data showing that low-dose VE therapy has a favorable risk profile and is minimally systemically absorbed.^8,16^ Randomized clinical trials (RCTs) have shown that low-dose VE tablets do not significantly increase serum estradiol levels, whereas the use of low-dose VE creams and rings results in a minimal increase in serum estradiol levels in some studies.^3^ The clinical meaning of these laboratory changes is unknown, and, to our knowledge, there are no RCTs powered to determine whether VE causes heart disease, cancer, or venous thromboembolism. However, observational data from the Women’s Health Initiative Study found no association between VE use and increased risk of stroke, cancer, or venous thromboembolism in 45 663 women followed for a median of 7 years.^8^ Furthermore, the authors observed lower risks of cardiovascular disease, fracture, and all-cause mortality among VE users than among nonusers.^8^

Despite evidence supporting the safety and efficacy of VE, contemporary clinical practice prescribing patterns for GSM remain unclear.^7,8^ To bridge this gap in knowledge, we analyzed Medicare outpatient and prescription claims data to evaluate VE prescriptions among postmenopausal US women with diagnoses indicative of GSM. We examined VE prescription claims across GSM symptom groups to identify which clinical phenotypes were more likely to fill a VE prescription.

## Methods

This cohort study was reviewed by the Stanford University institutional review board and was determined to be exempt from human participants review and informed consent. This report follows the Strengthening the Reporting of Observational Studies in Epidemiology (STROBE) reporting guidelines for cohort studies.

### Data Sources

This retrospective cohort study used data from a 20% random sample of Medicare Research Identifiable Files for beneficiaries enrolled between 2006 and 2018. The Medicare Research Identifiable Files database contains Master Beneficiary Summary Files, consisting of demographic data, and Medicare Provider Analysis and Review data, consisting of inpatient and skilled nursing facilities stays covered by Medicare Part A, outpatient visits covered by Medicare Part B, and prescription drug services covered by Medicare Part D.^17,18,19,20,21^

### Study Population

**Figure 1.**
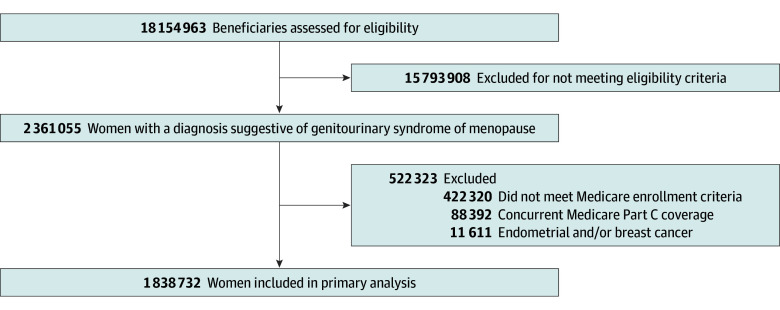
Participant Enrollment Flowchart Flowchart shows inclusion and exclusion criteria.

Among the 20% random sample, we constructed a cohort of female-identified beneficiaries with GSM, defined by having 1 or more GSM-related diagnosis (eTable 1 in Supplement 1). Women were included if they were aged 66 years or older and had continuous enrollment in Medicare parts A, B, and D for at least 1 year before and after their first GSM diagnosis to allow for a retrospective period to assess comorbidities and a prospective period to capture follow-up. Individuals with Medicare Advantage (Part C) and those with a diagnosis of breast and/or endometrial cancer within 6 months of their first GSM diagnosis were excluded because these are estrogen-sensitive cancers, and women may be advised to stop all estrogen therapies during treatment (Figure 1).

### Exposure Assessment

Exposure was defined as having a diagnosis indicative of GSM. GSM diagnoses were determined using codes from the *International Classification of Diseases, Ninth Revision (ICD-9-CM)* and the *International Statistical Classification of Diseases and Related Health Problems, Tenth Revision (ICD-10-CM* (eTable 1 in Supplement 1). Because GSM does not have a named *ICD-9* or *ICD-10* code, diagnoses were selected on the basis of the North American Menopause Society definition: genital symptoms of dryness, burning, and irritation; urinary symptoms and conditions of dysuria, urgency, and recurrent UTIs (rUTIs); and sexual symptoms of pain and dryness.^1,7^ On the basis of this framework, the selected diagnosis codes were then categorized into 5 distinct symptom groups: vulvovaginal (ie, vulvovaginal atrophy, pruritus, or vaginitis), bladder (ie, urinary urgency or frequency), urethral (ie, dysuria or urethral caruncle), local sexual (ie, dyspareunia, vulvodynia, or postcoital bleeding) and rUTI. rUTI was defined as 3 or more distinct UTI diagnoses, each 30 days or more apart, during the index diagnosis year. This classification system was developed to provide clarity on the association between the symptoms experienced by women with GSM and VE therapy utilization (eTable 1 in Supplement 1).

Although GSM does not have a named *ICD-9* or *ICD-10* code, the American Association of Professional Coders does recommend the use of the following codes for patients with GSM: N95.1 (menopausal and female climacteric states), N95.2 (postmenopausal atrophic vaginitis), N95.8 (other specified menopausal and perimenopausal disorders), N95.9 (unspecified menopausal and perimenopausal disorder), and the corresponding *ICD-9* codes (627.2, 627.3, 627.4, 627.8, and 627.9). Notably, these codes represent a subset of vulvovaginal diagnoses. To capture the effects of these coding recommendations, the vulvovaginal group was divided into 2 subgroups: those with 1 of the American Association of Professional Coders–recommended GSM diagnosis codes (specific GSM diagnosis) and those with the remaining vulvovaginal symptom diagnosis codes (other vulvovaginal diagnosis) (eTable 1 in Supplement 1).

### Outcomes

The primary study outcome was 1 or more VE prescription claim during the follow-up period. VE prescription fill was treated as a binary outcome (yes or no). The number of prescriptions filled per individual and the type of prescription (initial or refill) were not assessed. Follow-up began at the date of first GSM diagnosis and continued until the end of an individual’s Medicare enrollment or the end of the study period.

### Covariates

Collected variables included age, race and ethnicity, Medicare region, medical comorbidity, dual Medicare and Medicaid eligibility, formulation of VE prescription, VE prescriber taxonomy, and GSM diagnosis category. Race and ethnicity were obtained from the beneficiary enrollment file information and are included to enable examination of differences in prevalence, symptom presentation, and treatment utilization, serving as markers of social and structural determinants of health rather than inherent biological differences. Categories follow the classification system derived by the Research Triangle Institute and include American Indian or Alaska Native, Asian or Pacific Islander, Black, Hispanic, non-Hispanic White, other, and unknown; categories are mutually exclusive.^22^ Medicare region designation mirrors the 4 US census regions—West, Midwest, Northeast, and South. Charlson Comorbidity Index (CCI) was used to quantify medical comorbidity according to *ICD-9* and *ICD-10* codes. CCI is a validated tool intended to reflect the aggregate health of an individual and is used to adjust for confounding in epidemiologic research.^23^ Dual eligibility, defined as enrollment in both Medicare and Medicaid for at least 1 month within the index year, was used as a surrogate for socioeconomic status.^24^ National Drug Codes were used to identify all formulations of VE, including creams, tablets, and intravaginal rings (eTable 2 in Supplement 1). Prescriber information was consistently available between 2014 and 2018. Prescriber type (physician or advanced practice practitioner) and subspecialty were collected using National Provider Identification numbers.

### Statistical Analysis

Data analysis was performed from October 2023 to June 2024. VE utilization was defined as the proportion of women with any GSM-related diagnosis who filled 1 or more VE prescription. To quantify associations between patient characteristics and the likelihood of VE claims, we conducted univariable and multivariable logistic regression analyses. Univariable models estimated unadjusted odds ratios (ORs), whereas multivariable analysis adjusted for key covariates. Multivariable models were adjusted for age, race, region, and CCI. Missing data (race or region) were labeled as unknown. Because these covariates were not central to the study aims, all available data were included in the analyses without exclusion or imputation. Practitioner data were missing for many prescribers before 2014 and were, therefore, excluded from the main model. Dual-eligibility status was evaluated in univariable and sensitivity analyses to assess potential confounding and was excluded from the primary model, as it showed no association and did not meaningfully affect estimates.

To assess the impact of varying definitions of GSM and potential misclassification bias, a sensitivity analysis was performed excluding patients with bladder symptoms only, because these symptoms are considered the least specific to GSM and represented a large subset (20.8%) of our cohort. Fully adjusted ORs (aORs) were calculated on all observable patient characteristics. All statistical analyses were performed using SAS statistical software version 9.4 (SAS Institute). Two-sided *P* < .05 was considered statistically significant.

## Results

A total of 1 838 732 women with at least 1 GSM-related diagnosis were identified (median [IQR] age, 74 [69-81] years; 7851 American Indian or Alaska Native [0.4%], 39 062 Asian [2.1%], 141 558 Black [7.7%], 96 218 Hispanic [5.2%], and 1 534 763 non-Hispanic White [83.5%]) (Table 1). During the follow-up period (median [IQR], 8 [4-10] years), 165 530 women (9.0%) filled a prescription for VE. The median (IQR) time from GSM diagnosis to VE prescription fill was 15 (2-46) months. Between 2014 and 2018, gynecologists represented the highest proportion of prescribers (24 163 prescribers [38.0%]), followed by primary care physicians (21 576 prescribers [33.9%]), urologists (9160 prescribers [14.4%]), and advanced practice practitioners (7 976 prescribers [12.5%]). Vaginal creams were most frequently prescribed, representing 149 450 VE claims (90.3%).

**Table 1.** Baseline Characteristics of Beneficiaries With GSM of Menopause^a^

Characteristic	Beneficiaries, No. (%)		
	Total (N = 1 838 732)	Vaginal estrogen use	
		Yes (n = 165 530 [9.0%])	No (n = 1 673 202 [91.0%])
Age at earliest GSM diagnosis, y			
Mean (SD)	76 (8)	74 (7)	76 (8)
Median (IQR)	74 (69-81)	71 (68-78)	74 (69-82)
Age group, y			
66-70	650 474 (35.4)	74 489 (11.5)	575 985 (88.6)
71-75	381 720 (20.8)	37 364 (9.8)	344 356 (90.2)
76-80	298 284 (16.2)	25 225 (8.5)	273 059 (91.5)
81-85	246 624 (13.4)	16 540 (6.7)	230 084 (93.3)
≥86	261 630 (14.2)	11 912 (4.6)	249 718 (95.5)
Race^b^			
American Indian or Alaska Native	7851 (0.4)	644 (8.2)	7207 (91.8)
Asian or Pacific Islander	39 062 (2.1)	3606 (9.2)	35 456 (90.8)
Black	141 558 (7.7)	6897 (4.9)	134 661 (95.1)
Hispanic	96 218 (5.2)	10 178 (10.6)	86 040 (89.4)
Non-Hispanic White	1 534 763 (83.5)	142 445 (9.3)	1 392 318 (90.7)
Other^c^	11 600 (0.6)	885 (7.6)	10 715 (92.4)
Unknown^c^	7680 (0.4)	875 (11.4)	6805 (88.6)
Medicare region			
Northeast	250 417 (13.6)	22 728 (9.1)	227 689 (90.9)
Midwest	445 829 (24.3)	36 687 (8.2)	409 142 (91.8)
South	736 920 (40.1)	65 041 (8.8)	671 879 (91.2)
West	301 265 (16.4)	32 315 (10.7)	268 950 (89.3)
Unknown	104 301 (5.7)	8 759 (8.4)	95 542 (91.6)
Dual eligible status^d^	275 079 (15.0)	25 857 (9.4)	249 222 (90.6)
Charlson Comorbidity Index^e^			
0	645 463 (35.1)	69 733 (10.8)	575 730 (89.2)
1	408 401 (22.2)	39 150 (9.6)	369 251 (90.4)
2	272 862 (14.8)	22 808 (8.4)	250 054 (91.6)
3	191 520 (10.4)	14 350 (7.5)	177 170 (92.5)
4	114 229 (6.2)	7755 (6.8)	106 474 (93.2)
≥5	206 257 (11.2)	11 734 (5.7)	194 523 (94.3)
Symptom group^f^			
Vulvovaginal	984 001 (53.5)	139 838 (14.2)	844 163 (85.8)
Specific GSM diagnosis^g^	813 644 (44.3)	124 245 (15.3)	689 399 (84.7)
Other vulvovaginal diagnosis	170 357 (9.2)	15 593 (9.2)	154 764 (90.8)
Urethral	749 057 (40.7)	87 258 (11.7)	661 799 (88.3)
Bladder	1 098 446 (59.7)	108 677 (9.9)	989 769 (90.1)
Sexual	127 569 (6.9)	21 586 (16.9)	105 983 (83.1)
Recurrent UTI^h^	77 076 (4.2)	7813 (10.1)	69 263 (89.9)

Abbreviations: GSM, genitourinary syndrome of menopause; UTI, urinary tract infection.

^a^ GSM was defined as the presence of 1 or more of the 5 following categories of symptoms: vulvovaginal, urethral, bladder, sexual, and recurrent UTI.

^b^ Race and ethnicity data in Medicare claims are populated from Social Security Administration data; the categories are mutually exclusive.

^c^ Other and unknown are independent categories used by the Centers for Medicare and Medicaid Services.

^d^ Refers to individuals who had Medicare and Medicaid coverage for at least 1 month during the index year of GSM diagnosis.

^e^ This index is validated for use in administrative claims data (maximum score, 37).

^f^ Categories are not mutually exclusive.

^g^ Refers to American Association of Professional Coders–recommended GSM diagnosis codes.

^h^ Defined as 3 or more distinct UTI diagnoses, each 30 days or more apart, during the index diagnosis year.

The majority of the cohort (965 131 women [52.5%]) had conditions that fell into only 1 symptom group. The remainder of the cohort (873 601 women [47.5%]) experienced GSM multimorbidity (defined as the presence of multiple GSM symptom groups). The most frequent co-occurring GSM diagnosis groups are listed in Table 2. The top 11 symptom groups (5 isolated symptom groups and 6 symptom group combinations) represent 93.5% of the population (1 718 563 women), and each was included in the multivariable analysis. Each of the remaining combinations accounted for less than 1% of the cohort and were grouped together as other. Local sexual symptoms (ie, dyspareunia), the most commonly treated symptom group (21 586 women [16.9%]), was selected as the reference group.

**Table 2.** GSM Diagnosis Symptom Group Categories

Diagnosis category	Beneficiaries, No. (%)		
	Total	Vaginal estrogen use	
		Yes	No
Vulvovaginal only	386 273 (21.0)	30 593 (7.9)	355 680 (92.1)
Specific GSM diagnosis^a^	328 854 (17.9)	26 859 (8.2)	301 995 (91.8)
Other vulvovaginal diagnosis^b^	57 419 (3.1)	3734 (6.5)	53 685 (93.5)
Bladder only	383 158 (20.8)	8442 (2.2)	374 716 (97.8)
Urethral only	162 668 (8.9)	3561 (2.2)	159 107 (97.8)
Sexual only	17 895 (1.0)	443 (2.5)	17 452 (97.5)
Recurrent urinary tract infection only	15 137 (0.8)	204 (1.4)	14 933 (98.7)
Urethral plus bladder	211 234 (11.5)	10 055 (4.8)	201 179 (95.2)
Vulvovaginal plus bladder	209 750 (11.4)	28 359 (13.5)	181 391 (86.5)
Vulvovaginal plus urethral	102 753 (5.6)	13 950 (13.6)	88 803 (86.4)
Vulvovaginal plus sexual	21 510 (1.2)	3805 (17.7)	17 705 (82.3)
Vulvovaginal plus urethral plus bladder	182 759 (9.9)	42 220 (23.1)	140 539 (76.9)
Vulvovaginal plus urethral plus bladder plus sexual	25 426 (1.4)	8577 (33.7)	16 849 (66.3)

Abbreviation: GSM, genitourinary syndrome of menopause.

^a^ Refers to American Association of Professional Coders–recommended GSM diagnosis codes.

^b^ Other symptom groups accounted for the remaining 6.5% of the cohort (120 169 participants).

**Figure 2.**
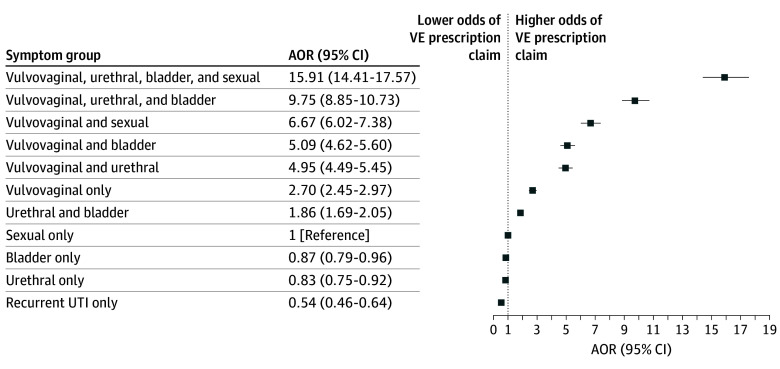
Association of Genitourinary Syndrome of Menopause Symptom Groups With the Likelihood of a Vaginal Estrogen (VE) Prescription Claim Local sexual symptoms served as the reference group. Logistic regression model controls for age, race, ethnicity, region, and Charlson Comorbidity Index. AOR indicates adjusted odds ratio; UTI, urinary tract infection.

In both the univariable and adjusted multivariable models, younger age was associated with a higher likelihood of VE prescribing, with each older age group being less likely to fill a prescription (aOR for age >86 years vs 66-70 years, 0.59; 95% CI, 0.58-0.60) (Table 3). Black (aOR, 0.60; 95% CI, 0.59-0.62), other (aOR, 0.79; 95% CI, 0.74-0.85), and American Indian or Alaska Native (aOR, 0.90; 95% CI, 0.82-0.97) racial groups were less likely than non-Hispanic White beneficiaries to fill a prescription. Healthier patients were more likely than less healthy patients to fill a prescription, with each incremental increase in CCI score corresponding to decreased odds of VE prescribing (aOR for score ≥5 vs 0, 0.67; 95% CI, 0.66-0.69) (Table 3). Patients with only vulvovaginal symptoms were most likely to fill a prescription compared with women with diagnoses in another single symptom group (aOR vs local sexual symptoms, 2.70; 95% CI, 2.45-2.97) (Figure 2; eTable 3 in Supplement 1). aORs were calculated for the vulvovaginal symptom subgroups specific GSM diagnosis and other vulvovaginal symptoms. Those with a specific GSM diagnosis (aOR, 2.75; 95% CI, 2.50-3.03) were slightly more likely than those with other vulvovaginal diagnoses (aOR, 2.41; 95% CI, 2.18-2.66) to fill a VE prescription. Bladder-only (aOR, 0.87; 95% CI, 0.79-0.96), urethral-only (aOR, 0.83; 95% CI, 0.75-0.92), and rUTI-only (aOR, 0.54; 95% CI, 0.46-0.64) symptoms were associated with the lowest odds of a VE prescription. Patients with GSM multimorbidity were more likely to have a prescription compared with patients with symptoms in 1 group (aOR, 15.91; 95% CI, 14.41-17.57). For example, in our adjusted model, those with vulvovaginal plus local sexual symptoms were 6.67 times more likely (95% CI, 6.02-7.38) to fill a prescription compared with women with local sexual symptoms only. Including dual eligibility as a covariate did not meaningfully change the aORs. In a sensitivity analysis in which patients with bladder symptoms only were excluded from the cohort, no notable differences in the odds of filling a VE prescription were observed (eTable 4 in Supplement 1).

**Table 3.** Multivariable Analysis of Association of Patient Characteristics With the Likelihood of a Vaginal Estrogen Prescription Claim

Variable	Adjusted OR (95% CI)^a^	*P* value
Age group, y (reference, 66-70 y)		
71-75	0.85 (0.84-0.86)	<.001
76-80	0.76 (0.75-0.77)	
81-85	0.68 (0.66-0.69)	
≥86	0.59 (0.58-0.60)	
Race (reference, non-Hispanic White)		
American Indian or Alaska Native	0.90 (0.82-0.97)	<.001
Asian or Pacific Islander	1.07 (1.03-1.11)	
Black	0.60 (0.59-0.62)	
Hispanic	1.21 (1.19-1.24)	
Other^b^	0.79 (0.74-0.85)	
Unknown^b^	1.35 (1.26-1.45)	
Medicare Region (reference, Midwest)		
Northeast	1.11 (1.09-1.13)	<.001
South	0.98 (0.97-0.998)	
West	1.21 (1.19-1.23)	
Unknown	1.02 (0.99-1.05)	
Charlson Comorbidity Index (reference, 0)		
1	0.93 (0.92-0.94)	<.001
2	0.84 (0.82-0.85)	
3	0.78 (0.77-0.80)	
4	0.75 (0.73-0.77)	
≥5	0.67 (0.66-0.69)	

^a^ Logistic regression model controlled for age, race, ethnicity, region, and Charlson Comorbidity Index.

^b^ Other and unknown are independent categories used by the Centers for Medicare and Medicaid Services.

## Discussion

Low-dose VE is a safe and effective treatment for GSM, a condition that negatively impacts the well-being of a large, growing population of postmenopausal women.^2^ To our knowledge, this cohort study is the first to describe VE utilization among a large, national cohort of women aged 66 years and older with documented GSM-related diagnoses. Overall, 9.0% of our cohort had a VE claim over the study period, and all clinical phenotypes of GSM had low rates of VE treatment. These findings are generalizable to postmenopausal US women aged 66 years and older. Utilization patterns may differ in younger perimenopausal and postmenopausal women. Beneficiaries with local sexual symptoms (ie, dyspareunia) had the highest utilization rate at 16.9%. Patients with vulvovaginal symptoms, local sexual symptoms, and GSM multimorbidity were more likely to fill a VE prescription than patients with urinary symptoms, including rUTI. This discrepancy may represent knowledge gaps regarding clinical indications for VE therapy. Furthermore, VE preparations are labeled by the Food and Drug Administration as indicated for the treatment of vulvovaginal atrophy. Although the term *GSM* was introduced to replace *vulvovaginal atrophy* and reflect the full spectrum of urogenital symptoms, it is not yet universally adopted or defined. No standardized criteria exist for the minimum number or type of symptoms required for diagnosis. In our cohort, VE use was observed across all clinical phenotypes, highlighting the need for careful consideration when developing future diagnostic criteria, since overly strict thresholds may limit treatment access for a condition that remains underdiagnosed and undertreated.

Differences in VE use requiring further study in future studies were observed across patient demographics. Patients of younger age and with lower CCI scores were more likely to be treated with VE. Compared with non-Hispanic White women, American Indian or Alaska Native and Black beneficiaries were less likely to have a VE claim. VE creams (90.3%) were most commonly prescribed, although previous trials suggest that both rings and tablets are preferred by patients.^25,26^

Our results echo several other studies that show low utilization of menopausal hormone therapies (MHTs).^9,27,28,29^ Weissfeld et al used claims data to show a 2.7% to 4.2% annual prevalence for VE products among postmenopausal women between 2006 and 2015.^9^ National survey data from a cohort of over 13 000 women showed that the prevalence of MHT, including systemic and low-dose vaginal preparations, declined from 26.9% in 1999 to only 4.7% in 2020.^27^ Declines in MHT are often attributed to the initial interpretation of the Women’s Health Initiative study released in 2002, which suggested that the risks of systemic MHT exceeded the benefits when used for chronic disease prevention in postmenopausal women.^30,31^

In our study, we analyzed VE use among postmenopausal women with GSM-related diagnoses. Low-dose VE has distinct indications and a different risk profile from systemic MHT, making it important to understand VE-specific utilization patterns. To our knowledge, no RCTs have specifically investigated associations of VE with cardiovascular disease, venous thromboembolism, cancer, or mortality. Reported adverse events are generally local, such as vaginal discharge.^32,33^ Most VE trials evaluate only a 12-week course, even though GSM is a progressive condition and requires long-term management. Safety data beyond 1 year of VE use remain limited, highlighting the need for further research on long-term outcomes.^33,34^

In this study, we excluded patients with diagnoses of breast and/or endometrial cancer within 6 months of their first GSM diagnosis. There are limited clinical trial data demonstrating safety of VE in individuals with prior diagnoses of breast and/or endometrial cancers, although a growing number of observational studies suggest that it may be safe.^35,36,37,38^ In patients with a history of breast cancer, large observational studies have shown no increase in risk of recurrence with VE, with the exception of a subgroup receiving both low-dose VE and aromatase inhibitors.^35,36,37^ One RCT of women with a history of stage I or II endometrial cancer treated with oral estrogen or placebo had low overall cancer recurrence rates, suggesting that low-dose VE may also be low risk.^39^ In practice, low-dose VE may be prescribed in patients with a history of estrogen-sensitive cancers in the context of individualized, multidisciplinary shared decision-making.^15,40^

Understanding the impact of GSM on the genitalia and lower urinary tract is critical for all practitioners caring for postmenopausal women. Standardized diagnostic criteria, patient-practitioner education, and an optimized coding schema for GSM could improve identification and study of this condition. Current American Association of Professional Coders codes are nonspecific and omit local sexual and lower urinary tract symptoms. We propose a specific *ICD-10* diagnosis code for GSM that distinguishes vulvovaginal, local sexual, and urinary symptoms, analogous to codes for benign prostatic hyperplasia, which differentiate cases with and without lower urinary tract symptoms (*ICD-10* codes N40.0 vs N40.1).

### Strengths and Limitations

A strength of this study is its use of a large, population-based Medicare dataset, providing nationally representative estimates for older US adults and sufficient statistical power to detect differences across clinical subgroups. Limitations of our study include the absence of granular data impacting decision-making for VE therapy. Our cohort was selected using *ICD-9* and *ICD-10* codes that do not capture symptom severity, clinician decision-making, patient preferences, or access to care, all of which may impact VE prescribing patterns. We sought to mitigate this by, for example, using strict rUTI criteria. Furthermore, alternative GSM therapies, including vaginal dehydroepiandrostone, ospemifene, vaginal moisturizers, and symptom-directed therapies, were not assessed. Vaginal dehydroepiandrostone (Food and Drug Administration approved in 2016) and ospemifene (Food and Drug Administration approved in 2013) were excluded because these treatments were not commercially available for the entirety of the study period. These gaps limit assessment of VE appropriateness and preclude defining a target utilization rate.

Our data may underestimate VE prescribing because of several factors. VE prescriptions obtained from alternative sources (ie, Medicare Advantage or online pharmacies) were not captured. Gaps in Medicare part D coverage after 12 months of continuous enrollment may have affected coverage of VE for some patients and, therefore, the likelihood of a prescription claim. We defined GSM broadly, although patients with a specific GSM diagnosis also had low utilization rates. Follow-up time varied, although the median duration was 8 years, reducing the risk that differential follow-up biased the likelihood of observing a prescription claim.

## Conclusions

In this large, national cohort study of female Medicare beneficiaries with GSM, only 9.0% of beneficiaries filled a VE prescription. Most women with GSM symptoms, including vulvovaginal atrophy, dyspareunia, and rUTI, did not fill a VE prescription. Younger and healthier beneficiaries and those with GSM multimorbidity were more likely to have a VE claim. Future efforts should optimize coding and validate our proposed GSM phenotypes in administrative data to better identify symptomatic women, characterize clinically meaningful and resource-intensive phenotypes, enhance patient-practitioner education, track therapy use, assess outcomes, and evaluate the population-level impact of new interventions to improve patient care.

## Notes

Supplement 1. eTable 1. *International Classification of Diseases, Ninth Revision (ICD-9)* and the *International Statistical Classification of Diseases and Related Health Problems, Tenth Revision (ICD-10)* Codes Used to Define Patients With Genitourinary Syndrome of Menopause eTable 2. National Drug Codes (NDC) Used to Identify All Formulations of VE Including Vaginal Creams, Vaginal Tablets and Intravaginal Rings eTable 3. Multivariable Analysis of GSM Symptom Group on the Likelihood of a Vaginal Estrogen Prescription Claim eTable 4. Multivariable Analysis of Patient Characteristics on the Likelihood of a Vaginal Estrogen Prescription Claim: Sensitivity Analysis Excluding Patients With Bladder Symptoms Only

Supplement 2. Data Sharing Statement
