# Supplementary material for: Vaginal Estrogen Utilization Among Medicare Beneficiaries With Genitourinary Syndrome of Menopause
Source: JAMA Netw Open. 2025 Dec 16;8(12):e2549822. doi: 10.1001/jamanetworkopen.2025.49822 (PMC12709372; doi:10.1001/jamanetworkopen.2025.49822)
Supplement: Supplement 1. — eTable 1. International Classification of Diseases, Ninth Revision (ICD-9) and the International Statistical Classification of Diseases and Related Health Problems, Tenth Revision (ICD-10) Codes Used to Define Patients With Genitourinary Syndrome of Menopause eTable 2. National Drug Codes (NDC) Used to Identify All Formulations of VE Including Vaginal Creams, Vaginal Tablets and Intravaginal Rings eTable 3. Multivariable Analysis of GSM Symptom Group on the Likelihood of a Vaginal Estrogen Prescription Claim eTable 4. Multivariable Analysis of Patient Characteristics on the Likelihood of a Vaginal Estrogen Prescription Claim: Sensitivity Analysis Excluding Patients With Bladder Symptoms Only [file jamanetwopen-e2549822-s001.pdf]

Supplemental Online Content

Gallo K, Burton C, Zhang CA, Kamdar N, Enemchukwu EA. Vaginal estrogen utilization among Medicare beneficiaries with genitourinary syndrome of menopause. *JAMA Netw Open*. 2025;8(12):e2549822. doi:10.1001/jamanetworkopen.2025.49822

eTable 1. *International Classification of Diseases, Ninth Revision (ICD-9)* and the *International Statistical Classification of Diseases and Related Health Problems, Tenth Revision (ICD-10)* Codes Used to Define Patients With Genitourinary Syndrome of Menopause
eTable 2. National Drug Codes (NDC) Used to Identify All Formulations of VE Including Vaginal Creams, Vaginal Tablets and Intravaginal Rings
eTable 3. Multivariable Analysis of GSM Symptom Group on the Likelihood of a Vaginal Estrogen Prescription Claim
eTable 4. Multivariable Analysis of Patient Characteristics on the Likelihood of a Vaginal Estrogen Prescription Claim: Sensitivity Analysis Excluding Patients With Bladder Symptoms Only

This supplemental material has been provided by the authors to give readers additional information about their work.

© 2025 Gallo K et al. *JAMA Network Open*.

eTable 1. *International Classification of Diseases, Ninth Revision (ICD-9)* and the *International Statistical Classification of Diseases and Related Health Problems, Tenth Revision (ICD-10)* Codes Used to Define Patients With Genitourinary Syndrome of Menopause.

		ICD-9	ICD-10
Vulvovaginal Symptoms	Specific GSM diagnosis	627.2 Symptomatic menopausal or female climacteric states<br>627.3 Postmenopausal atrophic vaginitis<br>627.4 Symptomatic states associated with artificial menopause<br>627.8 Other specified menopausal and postmenopausal disorders<br>627.9 Unspecified menopausal and post-menopausal disorder	N95.1 Menopausal and female climacteric states<br>N95.2 Postmenopausal atrophic vaginitis<br>N95.8 Other specified menopausal and perimenopausal disorders<br>N95.9 Unspecified menopausal and perimenopausal disorder
	Other vulvovaginal diagnosis	616.10 Vaginitis and vulvovaginitis unspecified<br>623.2 Vaginal adhesions/stenosis/occlusion/stricture<br>623.5 Noninfectious vaginal leukorrhea<br>623.8 Nonspecific inflammatory disorder of vagina<br>624.1 Atrophy of vulva<br>625.71 Vulvar vestibulitis	N90.5 Atrophy of vulva<br>N94.810 Vulvar vestibulitis<br>N76.1 Subacute and chronic vaginitis<br>N76.0 Acute vaginitis<br>N76.89 Other specified inflammation of vagina and vulva<br>L29.2: Pruritus vulvae<br>N89.5 Vaginal adhesions/stenosis/occlusion/stricture<br>N89.8 Other non-inflammatory disorders of the vagina
Sexual Symptoms		625.0 Dyspareunia<br>625.1 Vaginismus<br>625.79 other vulvodynia<br>625.70 Vulvodynia, unspecified<br>626.7 Postcoital bleeding<br>302.73 Female orgasmic disorder	N94.10 Unspecified dyspareunia<br>N94.11 Superficial (introital) dyspareunia<br>N94.12 Deep dyspareunia<br>N94.19 Other specified dyspareunia<br>N94.2 Vaginismus<br>N94.81 Vulvodynia<br>N94.818 Other vulvodynia<br>N94.819 Vulvodynia unspecified<br>R102 Pelvic and perineal pain

© 2025 Gallo K et al. *JAMA Network Open*.

		N93.0 Postcoital and contact bleeding<br>F52.31: Female orgasmic disorder
Bladder Symptoms	788.30 Unspecified urinary incontinence<br>788.31 Urge incontinence<br>788.33 Mixed incontinence<br>788.34 Incontinence without sensory awareness<br>788.36 Nocturnal enuresis<br>788.39 Other urinary incontinence<br>788.41 Urinary frequency<br>788.42 polyuria<br>788.43 nocturia<br>788.63 urgency of urination<br>788.91 Functional urinary incontinence<br>596.51 Hypertonicity of bladder/overactive bladder<br>596.59 Detrusor instability<br>596.89 Other specified disorders	R32 Urinary incontinence unspecified<br>R32.81 Overactive bladder<br>N39.41 Urge incontinence<br>N39.46 Mixed incontinence<br>N39.42 Incontinence without sensory awareness<br>N39.44 Nocturnal enuresis<br>N39.49 Other specified urinary incontinence<br>R35 Frequency of micturition<br>R35.1 Nocturia<br>R35.8 Other polyuria<br>R39.15 Urgency of urination<br>R39.81 Functional urinary incontinence<br>F98.0 Nonorganic enuresis
Urethral Symptoms	788.1 Dysuria<br>597.80 Urethritis unspecified<br>597.81 Urethral syndrome not otherwise specified<br>597.89 Other urethritis, post menopausal<br>599.3 Urethral caruncle	R30.0 Dysuria<br>N36.2 Urethral caruncle<br>N34.1 Nonspecific urethritis<br>N34.2 Other urethritis, post menopausal urethritis<br>N34.3 Urethral syndrome, unspecified<br>N34.2 Other urethritis, post menopausal urethritis
Recurrent UTI diagnoses	V13.02 Personal history, urinary tract infection<br>599.0 Urinary tract infection, site not specified<br>595.0 Acute cystitis<br>595.3 Trigonitis<br>595.89 Other specified types of cystitis<br>595.9 Cystitis, unspecified without hematuria<br>590.1 Acute pyelonephritis<br>590.10 Acute pyelonephritis without lesion of renal medullary necrosis	Z87.440 Personal history of urinary tract infections<br>N39.0 Urinary tract infection, site not specified<br>N30.00 Acute cystitis without hematuria<br>N30.01 acute cystitis with hematuria<br>N30.20 Other chronic cystitis without hematuria<br>N30.21 Other chronic cystitis with hematuria<br>N30.30 Trigonitis without hematuria<br>N30.31 Trigonitis with hematuria

© 2025 Gallo K et al. *JAMA Network Open*.

	590.11 Acute pyelonephritis with lesion of renal medullary necrosis<br>590.80 Pyelonephritis, unspecified	N30.80 Other cystitis without hematuria<br>N30.81 Other cystitis with hematuria<br>N30.90 Cystitis, unspecified without hematuria<br>N30.91 Cystitis, unspecified with hematuria<br>N10 Acute pyelonephritis

© 2025 Gallo K et al. *JAMA Network Open*.

eTable 2. National Drug Codes (NDC) Used to Identify All Formulations of VE Including Vaginal Creams, Vaginal Tablets and Intravaginal Rings.

0430-3754 Estrace
50090-2321 Estrace
50090-3645 Estradiol
0093-3223 Estradiol
0093-3541 Estradiol
0115-1518 Estradiol
0378-8770 Estradiol
45802-097 Estradiol
47781-104 Estradiol
53746-226 Estradiol
0046-0872 Premarin
0169-5176 Vagifem
54868-6182 Vagifem
42291-962 Yuvafem
42291-426 Yuvafem
65162-226 Yuvafem
50261-104 Imvexxy
50261-110 Imvexxy
0013-2150 Estring
54868-5538 Estring

© 2025 Gallo K et al. *JAMA Network Open*.

eTable 3. Multivariable Analysis of GSM Symptom Group on the Likelihood of a Vaginal Estrogen Prescription Claim

GSM Symptom Group (vs Sexual only [REF])	Adjusted Odds Ratio[a] (95% CI)
Recurrent UTI only	0.54 (0.46 - 0.64)
Urethral only	0.83 (0.75 - 0.92)
Bladder only	0.87 (0.79 - 0.96)
Urethral+Bladder	1.86 (1.69 - 2.05)
Vulvovaginal only	2.70 (2.45 - 2.97)
Specific GSM diagnosis[b]	2.41 (2.18 - 2.66)
Other vulvovaginal diagnosis	2.75 (2.50 - 3.03)
Vulvovaginal+Urethral	4.95 (4.49 - 5.45)
Vulvovaginal+Bladder	5.09 (4.62 - 5.6)
Vulvovaginal+Sexual	6.67 (6.02 - 7.38)
Vulvovaginal+Urethral+Bladder	9.75 (8.85 - 10.73)
Vulvovaginal+Urethral+Bladder+Sexual	15.91 (14.41 - 17.57)

Abbreviations: GSM, genitourinary syndrome of menopause; CI, confidence interval; UTI, urinary tract infection

[a]Logistic regression model controlling for age, race/ethnicity, region and Charlson comborbidity index

[b]AAPC (American Association of Professional Coders) recommended GSM diagnosis codes

*****Others v Sexual only, aOR 5.76 (5.23 - 6.34)

© 2025 Gallo K et al. *JAMA Network Open*.

eTable 4. Multivariable Analysis of Patient Characteristics on the Likelihood of a Vaginal Estrogen Prescription Claim: Sensitivity Analysis Excluding Patients With Bladder Symptoms Only.

Variable	Adjusted Odds Ratio[a] (95% CI)	p-value
Age in years (vs 66-70 [REF])		<0.001
71-75	0.85 (0.84 - 0.87)	
76-80	0.77 (0.76 - 0.79)	
81-85	0.70 (0.68 - 0.71)	
≥86	0.64 (0.62 - 0.65)	
Race (vs Non-Hispanic White [REF])		<0.001
American Indian/Alaska Native	0.90 (0.83 - 0.98)	
Asian/Pacific Islander	1.09 (1.05 - 1.13)	
Black	0.61 (0.59 - 0.63)	
Hispanic	1.21 (1.18 - 1.24)	
Others	0.79 (0.73 - 0.85)	
Unknown	1.35 (1.25 - 1.46)	
Medicare Region (vs Midwest [REF])		<0.001
Northeast	1.12 (1.10 - 1.14)	
South	0.98 (0.96 - 0.99)	
West	1.20 (1.18 - 1.22)	
Unknown	1.02 (0.99 - 1.05)	
Charlson comorbidity index (vs 0 [REF])		<0.001
1	0.93 (0.92 - 0.94)	
2	0.84 (0.83 - 0.86)	
3	0.79 (0.77 - 0.81)	
4	0.76 (0.74 - 0.78)	
≥5	0.69 (0.67 - 0.70)	
GSM Symptom Group (vs Sexual only [REF])		<0.001
Recurrent UTI only	0.54 (0.46 - 0.64)	
Urethral only	0.84 (0.76 - 0.93)	
Urethral+Bladder	1.89 (1.72 - 2.09)	
Vulvovaginal only	2.77 (2.52-3.05)	
Vulvovaginal+Urethral	5.10 (4.63 - 5.63)	
Vulvovaginal+Bladder	5.24 (4.76 - 5.77)	
Vulvovaginal+Sexual	6.84 (6.18 - 7.57)	
Vulvovaginal+Urethral+Bladder	10.06 (9.14 - 11.08)	
Vulvovaginal+Urethral+Bladder+Sexual	16.44 (14.89 - 18.16)	

Abbreviations: GSM, genitourinary syndrome of menopause; CI, confidence interval; UTI, urinary tract infection
[a]Logistic regression model controlling for age, race/ethnicity, region and Charlson comborbidity index

*Others v Sexual only, aOR 5.16 (4.69 - 5.69)

© 2025 Gallo K et al. *JAMA Network Open*.
