# Supplementary material for: Vaginal Estrogen Utilization Among Medicare Beneficiaries With Genitourinary Syndrome of Menopause
Source: JAMA Netw Open. 2025 Dec 16;8(12):e2549822. doi: 10.1001/jamanetworkopen.2025.49822 (PMC12709372; doi:10.1001/jamanetworkopen.2025.49822)
Supplement: Supplement 2. — Data Sharing Statement [file jamanetwopen-e2549822-s002.pdf]

Data Sharing Statement

Gallo. Vaginal Estrogen Utilization Among Medicare Beneficiaries With Genitourinary Syndrome of Menopause. *JAMA Netw Open*. Published December 16, 2025. doi:10.1001/jamanetworkopen.2025.49822

Data
Data available: No

Additional Information
Explanation for why data not available: According to Medicare DUA, this data cannot be shared.
